# Medication adherence trajectory and its impact on recurrent stroke after carotid artery stenting

**DOI:** 10.3389/fneur.2025.1637268

**Published:** 2025-09-25

**Authors:** Shou-hong Lin, Mei-ling Yang, Fei-fei Liu, Yi Zeng, Ting-Ting Wu

**Affiliations:** ^1^Department of Neurology, The First Affliated Hospital, Fujian Medical University, Fuzhou, China; ^2^Department of Neurology, National Regional Medical Center, Binhai Campus of the First Affliated Hospital, Fujian Medical University, Fuzhou, China; ^3^Department of Nursing, The First Affliated Hospital, Fujian Medical University, Fuzhou, China

**Keywords:** carotid artery stenting, medication adherence, group-based trajectory model, stroke recurrence, Kaplan-Meier curves

## Abstract

**Background:**

Medication adherence is critical for patient outcomes, but remains insufficiently investigated in individuals following Carotid Artery Stenting (CAS). This study aims to investigate the trajectories and determinants of medication adherence and their impact on stroke recurrence in post-CAS patients.

**Methods:**

This study included patients who underwent CAS in the neurology department of a tertiary Grade A hospital in Fujian Province from January 2021 to May 2024. Medication adherence was assessed using the Medication Adherence Scale (MMAS-8) at 3, 6, and 12 months post-discharge, Stroke recurrence was evaluated at each follow-up visit. Group-Based Trajectory Modeling (GBTM) was utilized to analyze adherence trajectories, and logistic regression identified associated factors. Kaplan-Meier curves and Cox proportional hazards models were employed to assess the impact of adherence trajectories on stroke recurrence. Three Cox models were constructed: Model 1 (unadjusted), Model 2 (adjusted for age and sex), and Model 3 (further adjusted for significant variables from univariate analysis).

**Results:**

Medication adherence scores at 3, 6, and 12 months were 6.33 ± 1.43, 5.06 ± 1.31, and 4.81 ± 1.47, respectively. Three distinct adherence trajectories were identified: high level decline (37.32%), medium level decline (31.58%), and persistently low level decline (31.10%). Multivariate regression analysis revealed that household income, family medication supervision, regular follow-up visits, and medication related-beliefs significantly influenced these trajectories (*P* < 0.05). The 12-month stroke recurrence rate was 9.57%. Kaplan-Meier analysis demonstrated significant differences in stroke recurrence among the three trajectory groups (Log-rank and Breslow test, both *P* < 0.001). In Models 1 and 2, high level decline was associated with significantly lower recurrence risk compared with persistently low level decline (*P* < 0.05), but this association was not significant in Model 3 (*P* > 0.05).

**Conclusion:**

Post-CAS patients demonstrate three distinct medication adherence trajectories, with socioeconomic, behavioral, and belief-related factors influencing long-term adherence. The high level decline group serves as a crucial protective factor in preventing stroke recurrence. Underscoring the need for targeted interventions to maintain adherence and prevent recurrent stroke.

## 1 Introduction

Cerebrovascular disease remains the leading cause of mortality worldwide, with carotid artery stenosis being a major independent risk factor for cerebral infarction, accounting for approximately 20% of cases Carotid artery stenting (CAS) has emerged as a well-established therapeutic option for carotid artery stenosis (1), particularly for patients at elevated surgical risk (2). After carotid stent implantation, long-term antiplatelet therapy is essential to prevent stent thrombosis and subsequent ischemic events (3). However, treatment efficacy is significantly compromised by poor medication adherence or early discontinuation (4). Numerous studies have shown that medication adherence after CAS correlates with subsequent adverse events, including readmission rates and cardiovascular complications (5–7). Medication non-adherence is a pervasive global issue. In developed countries, non-adherence rates for chronic conditions reach up to 50%, with even higher rates in resource-limited settings (8). The consequences are profound, contributing to an estimated 125,000 deaths, 10% of hospitalizations, and $100–500 billion in annual healthcare costs in the United States alone (8). Recognizing its critical impact, multiple clinical guidelines have classified improving medication adherence as a Class I recommendation for chronic disease management, emphasizing its critical role in optimizing treatment outcomes and reducing adverse events (9, 10).

Managing medication adherence is inherently complex, as it is a dynamic process that fluctuates over time and is influenced by multiple factors. A study on adherence trajectories in patients with chronic diseases identified six distinct patterns: consistent adherence, persistent non-adherence, declining adherence, improving adherence, fluctuating adherence, and moderate adherence (11). Medication adherence is influenced by multiple factors, including patient-related factors, healthcare system and team-related factors, socioeconomic factors, condition-related factors, and therapy-related factors. However, the supporting evidence remains of low to moderate quality (11). Studies have demonstrated significant heterogeneity in the effectiveness of adherence-enhancing interventions, such as educational interventions, healthcare team-based strategies, and cognitive behavioral and motivational approaches, across different populations and time periods (8). Despite growing awareness of adherence trajectories in chronic disease management, evidence regarding postoperative medication adherence in CAS patients remains scarce. First, research predominantly focuses on drug efficacy and its effects on short-term clinical outcomes (12–14). Second, studies assessing medication adherence typically emphasize the perioperative period, lacking long-term follow-up and insights into adherence trajectories (6, 15). Third, the exploration of factors influencing adherence and potential interventions is limited. Existing literature indicates that gender and race are associated with postoperative medication adherence, highlighting the significance of institutional performance metrics, electronic health record reminders, and discharge prescription evaluations (6, 15). Lastly, current assessment methods often rely on prescription data and carotid artery revascularization and endarterectomy registry (CARET), which inadequately capture actual medication-taking behavior (6, 12, 15). Therefore, identifying adherence trajectories and their determinants following CAS, along with developing targeted interventions, is essential for optimizing adherence and improving long-term outcomes.

Given the research gap in post-CAS medication adherence, comprehensive studies are needed to examine its evolution and prognostic impact. We conducted a prospective cohort study, assessing medication adherence at 3-, 6-, and 12-months post-discharge and tracking 12-month stroke recurrence. Our objectives were to: (1) identify adherence trajectories using Group-Based Trajectory Modeling (GBTM); (2) determine influencing factors, including medication beliefs and social support; (3) evaluate their association with stroke recurrence. Findings will facilitate the identification of distinct medication adherence trajectories, inform targeted interventions, and enhance long-term outcomes in CAS patients.

## 2 Methods

### 2.1 Participants

This study utilized a continuous enrollment approach, recruiting voluntary participants who met eligibility criteria in the neurology department of a tertiary grade A comprehensive hospital in Fujian Province, China, from January 2021 to May 2023. The total study duration spanned from January 2021 to May 2024, comprising two phases: (1) the enrollment phase (January 2021 to May 2023) and (2) a 12-month follow-up period for all participants (until May 2024). Data analysis was conducted following the conclusion of the follow-up in May 2024.

Inclusion criteria were as follows: (1) Age ≥ 18 years. (2) Diagnosis confirmed according to the Guidelines for the Diagnosis and Treatment of Carotid Artery Stenosis (1). (3) Underwent carotid artery stenting. (4) For patients with a history of stroke, no significant residual sequelae, with a modified Rankin Scale (mRS) score of 0–2 (16). Exclusion criteria encompassed: (1) Patients with cardioembolic sources and intracranial atherosclerosis (ICAS). (2) Patients with impaired consciousness and communication disorders who cannot cooperate with the investigation. (3) Patients with severe mental illness and psychological disorders. (4) Patients with significant cardiac, pulmonary, or renal dysfunction, severe liver damage, or malignant tumors. Dropout criteria were as follows: (1) Patients lost to follow-up. (2) Patients who withdrew from the study, including those unreachable after two consecutive contact attempts, with a 1-day interval between attempts.

### 2.2 Data collection tools and methods

#### 2.2.1 Exposure

The Medication Adherence Scale (MMAS-8): Originally developed by Morisky et al. (17) and translated by Wang et al. (18). This scale consists of 8 items, with the first 7 items scored as follows: 0 for “yes” and 1 for “no.” The scoring for the 8th item is as follows: “Never” scores 1, “Occasionally” scores 0.75, “Sometimes” scores 0.5, “Often” scores 0.25, and “Always” scores 0. The total score ranges from 0 to 8, with higher scores indicating better medication adherence. A total score of 8 signifies good adherence, scores between 6 and 7.9 indicate moderate adherence, while scores below 6 reflect poor adherence. The Cronbach's α coefficient for the Chinese version of the MMAS-8 is 0.65. (The use of this scale has been approved by the original authors.)

#### 2.2.2 Outcomes

Recurrent stroke: the diagnostic criteria for recurrent stroke (19) include new neurological deficits and signs despite stable or improved symptoms from the initial ischemic stroke. Confirmation of new ischemic lesions is required through cranial CT or MRI.

#### 2.2.3 Other covariates

a. General Information: a custom-made questionnaire was used to collected demographic data including gender, age, marital status, education level, healthcare payment methods, and disease-related data such as self-care abilities and comorbidities. The number of implanted stents was also recorded.

b. Medication Adherence Belief Scale: the Chinese version of the Beliefs about Medicines Questionnaire (BMQ) includes two subscales (20): Specific-Necessity and Specific-Concerns, Each dimension comprises 5 items, resulting in a total of 10 items assessed via a 5-point Likert scale ranging from “strongly disagree” 1 to “strongly agree” 5. Higher scores indicate greater perceived necessity or concerns regarding medication. The total medication beliefs score is derived by subtracting the concerns beliefs score from the necessity beliefs score, with higher values reflecting stronger medication-related beliefs. The Cronbach's α coefficients for the necessity and concerns dimensions were 0.81 and 0.71, respectively, demonstrating good reliability and validity.

c. The Social Support Rating Scale (SSRS): the Chinese version of the Social Support Rate Scale (SSRS) developed by Xiao (21), consisting of 10 items scored on a 4-point scale. Higher scores indicate greater social support. The scale demonstrates good internal consistency reliability with a coefficient of 0.72 and high test-retest reliability of 0.92.

#### 2.2.4 Data collection

Researchers adhered strictly to the inclusion and exclusion criteria for participant selection, conducting data collection only after obtaining informed consent from patients or their family members. General information, BMQ, and SSRS were collected through face-to-face questionnaires before discharge, while MMAS-8 was assessed via telephone or outpatient follow-ups at 3 months (T1), 6 months (T2), and 12 months (T3) post-discharge. During follow-up, stroke recurrence was assessed by querying clinical symptoms and reviewing electronic medical records or remote imaging data. Additionally, patients were asked whether they had regular follow-up visits. To ensure data validity, incomplete or invalid questionnaires were promptly excluded. Verified data were entered into a computer database, with cross-checking performed by two independent reviewers at the study's conclusion.

### 2.3 Data analysis

#### 2.3.1 Sample size

There is no universally accepted standard for determining sample size or specific calculation methods for Group-Based Trajectory Modeling (GBTM). However, when using the Bayesian Information Criterion (BIC) as the primary model selection criterion, a minimum sample size of 200 is recommended to ensure robust outcomes. To enhance accuracy and model stability, a larger sample of 400 or more is advised if feasible (22). Accordingly, this study sets the minimum sample size requirement at 200 participants.

#### 2.3.2 Statistical analysis

GBTM was used to analyze changes in medication adherence trajectories. Evaluation criteria included: (1) Avepp to ensure accurate group allocation, with higher values (>0.7) indicating more reliable assignment to trajectory groups. (2) Pj and πj for comparing trajectory distribution proportions; closer values suggest a better model fit. (3) BIC for model fit assessment, where lower values indicate a better fit of the model to the data. (4) ΔBIC to evaluate differences in model fit, with values >10 suggesting a significantly better-fitting model. (5) OCC for assessing classification accuracy, with higher values reflecting increased confidence in the classification of individuals into trajectory groups. (6) Relative entropy for classification accuracy, with values >0.8 indicating at least 90% accuracy in group assignments (23).

Data management and statistical analyses were conducted using SAS 9.4. Categorical variables are reported as *n* (%), with chi-square and Fisher's exact tests employed for between-group comparisons. *T*-tests were used for normally distributed quantitative data, while the Mann-Whitney U test was applied for non-normally distributed data. Univariate analysis identified statistically significant variables, which were then included in multivariate regression to identify factors affecting adherence trajectories. Kaplan-Meier survival analysis was performed to estimate recurrence rates, followed by the construction of three Cox proportional hazards models: Model 1 (unadjusted), Model 2 (adjusted for age and sex), and Model 3 (further adjusted for significant variables from univariate analyses, including household income, family medication supervision, regular follow-up visits, medication adherence beliefs, residence, and living situation, etc.). Model selection criteria included a significance level of 0.05 and an exclusion criterion of 0.1, with statistical significance defined as *P* < 0.05.

### 2.4 Quality control

This study is a single-center investigation that strictly adhered to standardized protocols for patient screening, data collection, and evaluation, employing uniform inclusion and exclusion criteria to minimize data variability. Comprehensive data were gathered, including demographic characteristics, comorbidities, the number of stents implanted, and follow-up assessments. This meticulous data collection facilitates precise adjustment for confounding factors during analysis. Personnel involved in data collection and evaluation underwent rigorous training and calibration, complemented by regular audits to ensure measurement consistency and accuracy. Furthermore, ongoing monitoring and quality control checks were implemented to uphold adherence to the study protocol and to promptly identify and rectify any deviations, thereby preserving the integrity of the collected data.

## 3 Results

### 3.1 Patient demographics

**Figure 1:**
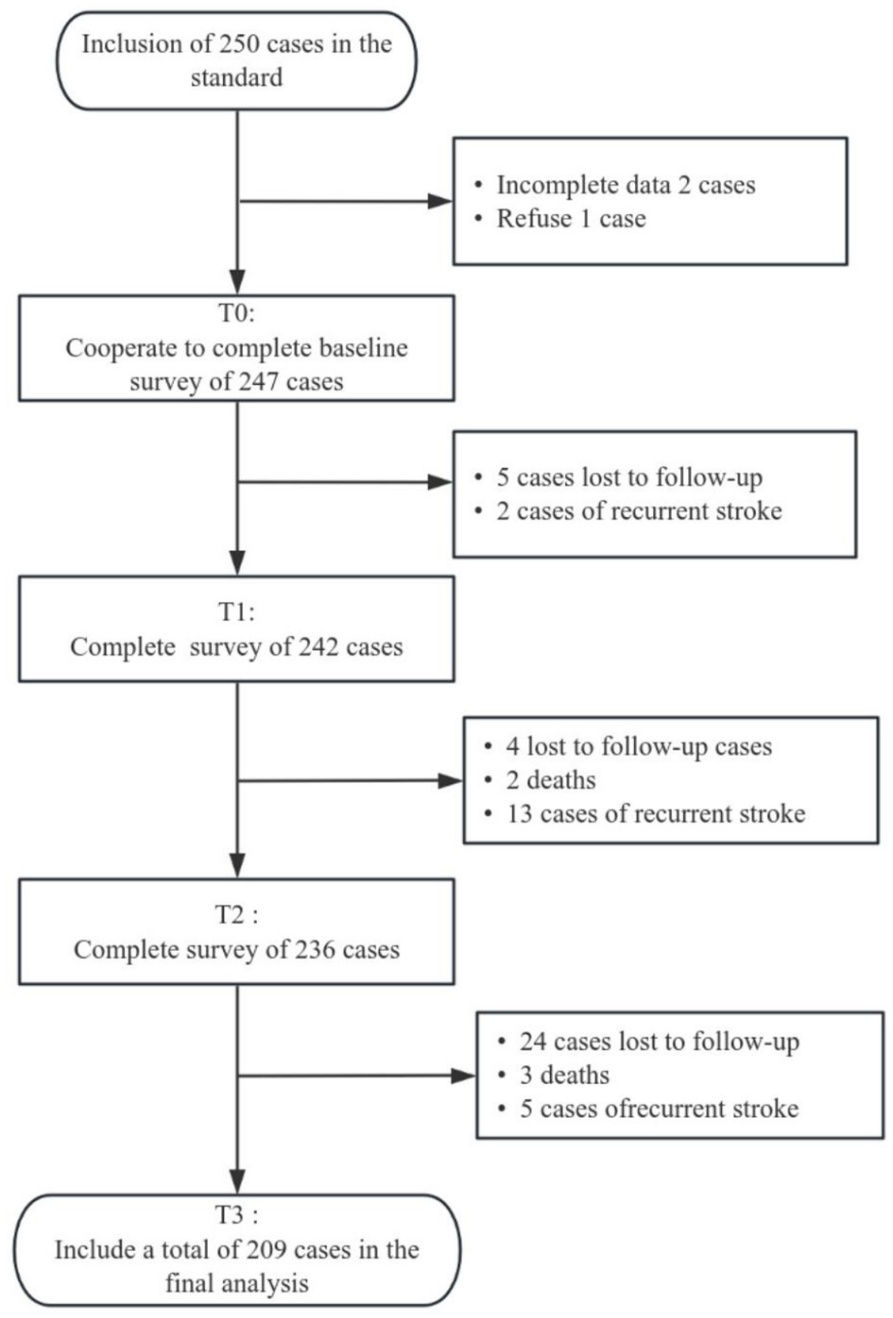
Subject Flowchart.

This study initially enrolled 250 participants, with 247 completing the baseline survey. Of these, 242 patients completed the T1 assessment, 236 completed the T2 assessment, and 209 completed the T3 assessment. Individuals lost to follow-up (5 at T1, 4 at T2, and 24 at T3) and those who died (2 at T2 and 3 at T3) were excluded from the analysis. Consequently, 209 participants with complete MMAS-8 data at all three time points were included in the GBTM analysis. Of these participants, 171 were male (81.82%) and 38 were female (18.18%); the average age was 66.8 ± 9.2 years. In terms of stent implantation, 110 cases (52.63%) had one CAS stent implanted, 61 cases (29.19%) had two, and 38 cases (18.18%) had three. Over 12-month follow-up, 20 cases of recurrent stroke were observed. Refer to Figure 1 and Table 1 for detailed data.

**Table 1:** Univariate analysis of different trajectories [*n*(%)].

**Variables**	**Total**	**Grouping**			**Statistic**	***P***
		**Persistently low level decline group (*****n*** = **65)**	**Medium level decline group (*****n*** = **66)**	**High level decline group (*****n*** = **78)**		
**Sex**						
Male	171 (81.82)	55 (84.62)	57 (86.36)	59 (75.64)	*χ^2^ =* 3.26	0.196
Female	38 (18.18)	10 (15.38)	9 (13.64)	19 (24.36)		
**Age (*****x*** **±*****s*****), years**	66.8 ± 9.2	65.8 ± 11.0	68.4 ± 9.0	66.3 ± 7.8	*F* = 1.46	0.235
**Age, years**						
< 60	49 (23.44)	18 (27.69)	12 (18.18)	19 (24.36)	*χ^2^ =* 3.01	0.556
60–70	81 (38.76)	25 (38.46)	24 (36.36)	32 (41.03)		
>70	79 (37.80)	22 (33.85)	30 (45.45)	27 (34.62)		
**Residence**						
Countryside	95 (45.45)	40 (61.54)	28 (42.42)	27 (34.62)	*χ^2^ =* 10.72	**0.005**
Urban area	114 (54.55)	25 (38.46)	38 (57.58)	51 (65.38)		
**Educational level**						
Junior high school and below	161 (77.03)	50 (76.92)	57 (86.36)	54 (69.23)	–	0.054^*^
High school or vocational school	33 (15.79)	13 (20.00)	5 (7.58)	15 (19.23)		
College degree or above	15 (7.18)	2 (3.08)	4 (6.06)	9 (11.54)		
**Occupation**						
Farmers	59 (28.23)	20 (30.77)	15 (22.73)	24 (30.77)	–	0.811^*^
Employees	16 (7.66)	5 (7.69)	6 (9.09)	5 (6.41)		
Individual business	7 (3.35)	2 (3.08)	2 (3.03)	3 (3.85)		
Leave, retire	57 (27.27)	13 (20.00)	22 (33.33)	22 (28.21)		
Others	70 (33.49)	25 (38.46)	21 (31.82)	24 (30.77)		
**Household income, yuan**						
< 3,000	44 (21.05)	23 (35.38)	12 (18.18)	9 (11.54)	*χ^2^ =* 24.61	**< 0.001**
3,000–6,000	81 (38.76)	30 (46.15)	26 (39.39)	25 (32.05)		
>6,000	84 (40.19)	12 (18.46)	28 (42.42)	44 (56.41)		
**Marital status**						
Married	194 (92.82)	61 (93.85)	61 (92.42)	72 (92.31)	–	1.000^*^
Divorced	4 (1.91)	1 (1.54)	1 (1.52)	2 (2.56)		
Other	11 (5.26)	3 (4.62)	4 (6.06)	4 (5.13)		
**Self-care abilities**						
Independent	109 (52.15)	38 (58.46)	32 (48.48)	39 (50.00)	*χ^2^ =* 1.54	0.464
Partially dependent	100 (47.85)	27 (41.54)	34 (51.52)	39 (50.00)		
**Living situation**						
With spouse	154 (73.68)	43 (66.15)	51 (77.27)	60 (76.92)	*χ^2^ =* 15.571	**0.004**
With children	30 (14.35)	6 (9.23)	12 (18.18)	12 (15.38)		
Non-family cohabitation	25 (11.96)	16 (24.62)	3 (4.55)	6 (7.69)		
**Healthcare payment methods**						
Medical insurance	199 (95.22)	60 (92.31)	63 (95.45)	76 (97.44)	–	0.369^*^
Self-pay	10 (4.78)	5 (7.69)	3 (4.55)	2 (2.56)		
**Hypertension**						
No	70 (33.49)	24 (36.92)	27 (40.91)	19 (24.36)	*χ^2^ =* 4.89	0.087
Yes	139 (66.51)	41 (63.08)	39 (59.09)	59 (75.64)		
**Diabetes**						
No	110 (52.63)	39 (60.00)	36 (54.55)	35 (44.87)	*χ^2^ =* 3.40	0.183
Yes	99 (47.37)	26 (40.00)	30 (45.45)	43 (55.13)		
**Heart disease**						
No	194 (92.82)	62 (95.38)	58 (87.88)	74 (94.87)	–	0.187^*^
Yes	15 (7.18)	3 (4.62)	8 (12.12)	4 (5.13)		
**Simultaneously accompanied by 2 or more of the above diseases**						
No	133 (63.64)	50 (76.92)	40 (60.61)	43 (55.13)	*χ^2^ =* 7.66	**0.022**
Yes	76 (36.36)	15 (23.08)	26 (39.39)	35 (44.87)		
**The number of implanted stents**						
1	110 (52.63)	43 (66.15)	33 (50.00)	34 (43.59)	*χ^2^ =* 15.82	**0.003**
2	61 (29.19)	20 (30.77)	17 (25.76)	24 (30.77)		
≥3	38 (18.18)	2 (3.08)	16 (24.24)	20 (25.64)		
**Family medication supervision**						
Yes	145 (69.38)	22 (33.85)	49 (74.24)	74 (94.87)	*χ^2^ =* 63.22	**< 0.001**
No	64 (30.62)	43 (66.15)	17 (25.76)	4 (5.13)		
**Regular follow-up visits**						
No	71 (33.97)	53 (81.54)	11 (16.67)	7 (8.97)	*χ^2^ =* 96.11	**< 0.001**
Yes	138 (66.03)	12 (18.46)	55 (83.33)	71 (91.03)		
Medication adherence belief (*x* ±*s*), points	9.92 ± 3.57	7.03 ± 2.89	9.88 ± 2.94	12.36 ± 2.66	*F* = 63.04	**< 0.001**
Social Support Scale (*x* ±*s*), points	38.4 ± 7.5	33.6 ± 6.8	38.8 ± 6.9	42.1 ± 6.2	*F* = 28.86	**< 0.001**

^*^Using Fisher's exact probability method. Items with statistically significant values are indicated in bold.

### 3.2 Medication adherence trajectories in CAS patients

Medication adherence scores at T1, T2, and T3 were 6.33 ± 1.43, 5.06 ± 1.31, and 4.81 ± 1.47, respectively, Medication adherence was analyzed as a continuous variable using GBTM with a censored normal distribution. Participants with fewer than three adherence measurements were excluded. Models with 1 to 4 groups were initially fitted using linear, quadratic, and cubic terms. The 4-group model was discarded as one trajectory group comprised less than 5% of the sample, resulting in the selection of a 3-group model. Based on trajectory plots and fit indices, the final model included three quadratic trajectories. See Supplementary Tables S1, S2.

**Figure 2:**
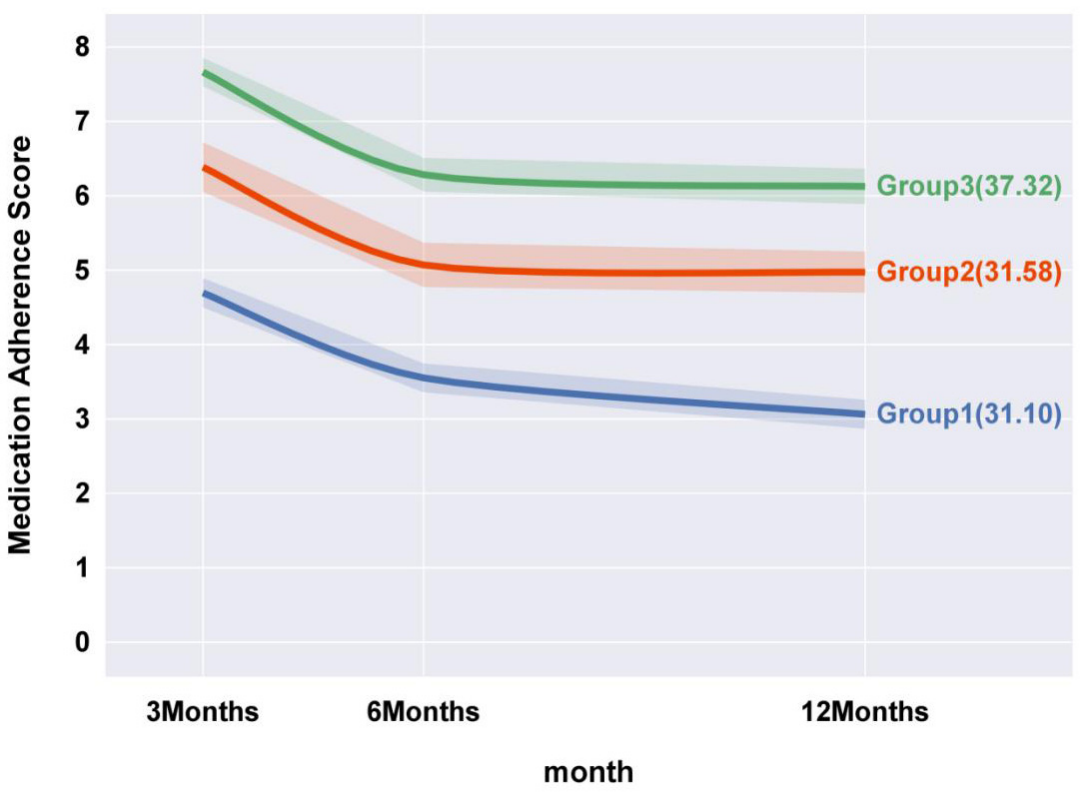
Medication adherence trajectory model for postoperative CAS patients.

This model categorizes patients into three distinct trajectories: Group 1, the “Persistently low level decline” cohort (31.10%), characterized by consistently low medication adherence throughout the study period, with a notable decline at 6 months, followed by a gradual decrease. Group 2, the “Medium level decline” cohort (31.58%), includes patients with initially moderate adherence, experiencing a sharp decline at 6 months before stabilizing. Group 3, the “High level decline” cohort (37.32%), represents patients with initially high adherence, undergoing a rapid decline at six months, followed by stabilization. See Figure 2.

### 3.3 Predictors of medication adherence trajectories

Multivariable regression analysis indicated monthly household income, family medication supervision, regular follow-up visits, and medication-related beliefs significantly influenced the trajectories of patients with CAS. Specifically, patients with a monthly household income exceeding 6,000 yuan were 17.562 times and 25.277 times more likely to transition to the medium level and high level decline groups, respectively, compared to those with an income below 3,000 yuan. Additionally, Patients without family supervision for medication adherence were less likely to transition to the high decline group, with an odds ratio of 0.07. respectively, compared to those with family supervision. Regular follow-up visits were associated with increased odds of transitioning to the medium level and high level decline groups, with odds ratios of 30.405 and 26.123, respectively, compared to patients with irregular follow-ups. Moreover, higher scores in medication beliefs were significantly associated with an increased likelihood of transitioning to both the medium level and high level decline groups, as shown in Tables 1, 2.

**Table 2:** Multifactor logistic analysis of different trajectories.

**Variable**	**Estimate**	**Standard error**	**Wald**	***P***	**OR (95%CI)**
**Medium level decline group vs. Persistently low level decline group**					
**Intercept**	−5.141	1.810	8.066	**0.005**	
**Household income, yuan**					
< 3,000					1.00 (ref)
3,000–6,000	0.230	0.728	0.100	0.752	1.258 (0.302–5.246)
>6,000	2.866	0.991	8.362	**0.004**	17.562 (2.518–122.50)
**Family medication supervision**					
Yes					1.00 (ref)
No	−0.974	0.590	2.723	0.099	0.378 (0.119–1.201)
**Regular follow-up visits**					
No					1.00 (ref)
Yes	3.415	0.730	21.896	**< 0.001**	30.405 (7.275–127.08)
**Medication adherence belief, points**	0.213	0.101	4.417	**0.036**	1.237 (1.014–1.508)
**Residence**					
countryside					1.00 (ref)
urban area	0.642	0.570	1.269	0.260	1.901 (0.622–5.810)
**Living situation**					
With spouse					1.00 (ref)
With children	0.028	0.774	0.001	0.971	1.028 (0.225–4.689)
Non-family cohabitation	−1.497	0.934	2.570	0.109	0.224 (0.036–1.395)
**Simultaneously accompanied by 2 or more of the above diseases**					
No					1.00 (ref)
Yes	0.392	0.677	0.335	0.563	1.479 (0.393–5.573)
**The number of implanted stents**					
1					1.00 (ref)
2	−0.987	0.686	2.067	0.151	0.373 (0.097–1.431)
≥3	2.174	1.550	1.968	0.161	8.796 (0.422–183.46)
**Social Support Scale, points**	0.028	0.044	0.422	0.516	1.029 (0.945–1.120)
**High level decline group vs. Persistently low level decline group**					
**Intercept**	−10.10	2.175	21.557	**< 0.001**	−10.10
**Household income, yuan**					
< 3,000					1.00 (ref)
3,000–6,000	0.302	0.869	0.121	0.728	1.352 (0.246–7.418)
>6,000	3.230	1.089	8.795	**0.003**	25.277 (2.990–213.69)
**Family medication supervision**					
Yes					1.00 (ref)
No	−2.665	0.792	11.327	**< 0.001**	0.070 (0.015–0.329)
**Regular follow-up visits**					
No					1.00 (ref)
Yes	3.263	0.861	14.361	**< 0.001**	26.123 (4.832–141.22)
Medication adherence belief, points	0.489	0.113	18.778	**< 0.001**	1.630 (1.307–2.033)
**Residence**					
Countryside					1.00 (ref)
Urban area	0.820	0.648	1.599	0.206	2.270 (0.637~8.083)
**Living situation**					
With spouse					1.00 (ref)
With children	−0.529	0.881	0.361	0.548	0.589 (0.105–3.308)
Non-family cohabitation	−0.475	1.099	0.187	0.666	0.622 (0.072–5.361)
**Simultaneously accompanied by 2 or more of the above diseases**					
No					1.00 (ref)
Yes	0.618	0.739	0.699	0.403	1.855 (0.436–7.894)
**The number of implanted stents**					
1					1.00 (ref)
2	−0.900	0.758	1.408	0.235	0.407 (0.092–1.798)
≥3	1.645	1.579	1.086	0.297	5.182 (0.235–114.35)
Social Support Scale, points	0.084	0.049	2.888	0.089	1.087 (0.987–1.197)

The model fitting index Hosmer-Lemeshow test χ^2^ = 13.23, P = 0.508, indicates that the model has a good fitting effect. Items with statistically significant values are indicated in bold.

### 3.4 Impact of medication adherence trajectories on 12-month stroke recurrence

**Figure 3:**
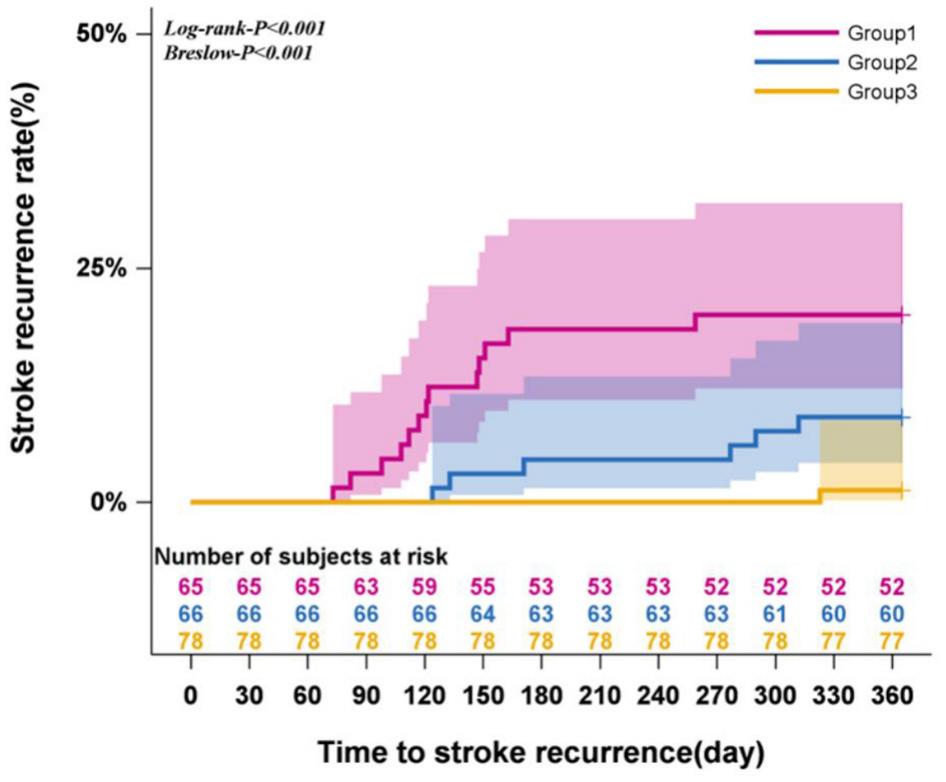
Survival analysis of recurrent stroke (Kaplan-Meier curve).

The 12-month stroke recurrence rate following CAS was 9.57%. Kaplan-Meier survival analysis indicated significant differences in stroke recurrence rates among the three adherence trajectory groups over time (Log-rank and Breslow test, both *P* < 0.001). Notably, with extended follow-up, the persistently low level decline group exhibited earlier recurrences and the most rapid increase in recurrence rates, indicating a higher risk compared to the high level decline group. In Cox proportional hazards models, both Model 1 and Model 2 analyses showed that patients in the high level decline group had a significantly lower risk of stroke recurrence compared to those in the persistently low level decline group (*P* < 0.05). Conversely, Model 3 revealed no significant association (*P* > 0.05). Detailed results are presented in Table 3 and Figure 3.

**Table 3:** Stepwise adjustment model analysis - Cox proportional hazards model.

**Group**	**Model 1**		**Model 2**		**Model 3**	
	**HR (95%CI)**	***P***	**HR (95%CI)**	***P***	**HR (95%CI)**	***P***
Model 1	1.00 (ref)		1.00 (ref)		1.00 (ref)	
Model 2	0.408 (0.155–1.074)	0.070	0.386 (0.145–1.028)	0.057	0.566 (0.125–2.559)	0.460
Model 3	0.056 (0.007–0.429)^*^	**0.005**	0.057 (0.007–0.436)^*^	**0.005**	0.499 (0.038–6.596)	0.598

Model 1 (null model), Model 2 (adjusted for age and sex), and Model 3 (further adjusted for household income, family medication supervision, regular follow-up visits, medication adherence beliefs, residence, living situation, simultaneous presence of two or more of the aforementioned diseases, number of implanted stents, and Social Support Scale).

^*^indicates *p* < 0.05. Items with statistically significant values are indicated in bold.

## 4 Discussion

To our knowledge, this is the first study to longitudinally characterize medication adherence trajectories, identify predictors, and assess the impact on stroke recurrence over 12 months in patients undergoing CAS. We identified significant heterogeneity in adherence trajectories, classifying patients into three groups: persistently low level decline (31.10%), medium level decline (31.58%), and high level decline (37.32%). Across different initial adherence levels, all groups experienced a rapid decline in the first 6 months, followed by either stabilization or further deterioration. Independent predictors of these trajectories included monthly household income, family medication supervision, regular follow-up visits, and medication related-beliefs. Notably, The high level decline group serves as a crucial protective factor in preventing stroke recurrence. Implementing a combined early risk prediction and stratified management approach to guide pharmacological intervention—especially during the initial 6 months post-surgery—may be critical for sustaining adherence and improving clinical outcomes.

### 4.1 Heterogeneity of medication adherence trajectories in CAS patients

Historical studies on medication adherence trajectories indicate significant variability across diseases and medications, with trajectory groups ranging from three to seven (11). For example, a study on statin adherence identified six trajectories: 21.1% of patients exhibited perfect or near-perfect adherence, while 19.5% were nearly completely nonadherent. The remaining 59.4% were classified as moderate adherers, with four subgroups: rapid decline, slow decline, occasional use, and brief gaps. Importantly, these statin adherence trajectories have been shown to predict future cardiovascular events (24). A cohort study on hypertension identified six medication adherence trajectories: 40% of patients were adherent, 10% experienced an early drop-off followed by a rebound to nearly full adherence, 10% exhibited partial drop-off, 14% showed gradual drop-off, 8% demonstrated rapid drop-off, and 18% had immediate drop-off. This underscores that medication adherence declines with an increase in the number of medications and treatment complexity (25). In a Chinese cohort of coronary artery disease patients (26), four adherence trajectories were identified: persistent adherence (39.9%), increasing then decreasing adherence (23.1%), increasing adherence (23.4%), and nonadherence (13.6%). Several factors may account for these discrepancies, including disease characteristics, treatment regimens, patient populations, and healthcare system differences.

Compared with more prevalent conditions such as hypertension and coronary artery disease, CAS is less common, and patients receive limited health education, which may contribute to lower medication awareness and a progressive decline in adherence over time. Additionally, differences in assessment tools may significantly impact study findings. This study utilized the MMAS-8 scale (27), which evaluates both medication-taking behavior and attitudes, and has been shown to perform well in identifying low adherence (28), whereas approximately two-thirds of adherence trajectory studies rely on proportion of days covered (PDC) metrics derived from pharmacy records (11). However, PDC is highly dependent on healthcare system structures, and in China, where patients do not consistently obtain medications from a single pharmacy, its reliability is limited. Furthermore, this study assessed adherence at 3, 6, and 12 months, which, while capturing overall trends, may not fully capture short-term fluctuations in adherence trajectories.

### 4.2 Factors influencing medication adherence trajectory in postoperative CAS patients

Medication adherence trajectories are shaped by multiple factors. Regular follow-up has been shown to reduce non-adherence (29), yet studies examining its impact on adherence trajectories are limited. Our analysis demonstrates that patients receiving regular follow-ups are 26.123 times more likely to fall into the high-level decline group, emphasizing the role of follow-up as a key predictor of adherence. Furthermore, we identify the lack of family supervision as a novel independent factor influencing adherence trajectories. The demographic characteristics of our cohort, including an average age of 66.8 ± 9.2 years and a prevalence of chronic comorbidities (82.8%), highlight the essential role of caregivers in medication management. Additionally, lower household income and negative medication beliefs independently affect adherence trajectories, aligning with findings in the existing literature (30, 31).

Independent factors influencing medication adherence trajectories after CAS provide critical opportunities for intervention in this patient population. First, evaluating financial barriers and medication beliefs shortly after discharge is practical and facilitates early identification of high-risk individuals. Second, incorporating structured caregiver involvement in medication management—through initial education and ongoing feedback—strengthens adherence oversight. Fourth, the incidence of mortality and loss to follow-up during the study led to a scarcity of data points, necessitating their exclusion from the analysis and obstructing a thorough evaluation of postoperative outcomes in CAS patients. Lastly, the single-center design might restrict the generalizability of these findings, highlighting the need for further research to address these limitations.

### 4.3 The association between medication adherence trajectories and stroke recurrence post-CAS surgery

This study investigates the association between medication adherence trajectories post-CAS and stroke recurrence, underscoring the importance of the high level decline group as a potential protective factor. The protective effect may stem from high medication adherence postoperatively, which is critical for thwarting stroke recurrence by inhibiting platelet aggregation and minimizing thrombotic risk (32, 33). While confounding variables hinder the establishment of a direct causal relationship, the findings endorse adherence trajectories as essential tools for clinical management. Prior research has established that low medication adherence is an independent predictor of adverse outcomes in chronic hepatitis B (CHB) and acute coronary syndrome (ACS) patients (34, 35). This study further affirms its clinical significance in preventing stroke recurrence following CAS.

The clinical implications of this study are as follows: clinicians should adopt early risk prediction and stratification approaches in their management strategies, informed by medication adherence trajectories post-CAS. First, medication adherence at 3 months post-procedure should be utilized to forecast subsequent trajectory patterns and stratify risk, thereby identifying high-risk populations based on influencing factors. Second, for patients in the persistently low adherence decline group, intensive interventions must be implemented to address medication-related concerns. This may include simplifying prescriptions based on financial constraints, providing low-cost alternatives, increasing follow-up frequency, incorporating electronic monitoring systems, and engaging caregivers in the intervention process. For the moderate decline group, it is essential to enhance positive behaviors through adherence reward programs and identify barriers. Patients in the high-level decline group should receive ongoing support, with a focus on reinforcing necessary beliefs to mitigate burnout. Finally, intensifying interventions within the first 6 months postoperatively is critical to preventing declines in adherence.

This study has several limitations. First, we assessed medication adherence among CAS patients at only three time points, which precluded a detailed understanding of the trajectory curves. Second, future studies should increase the sample size and incorporate potential mediating variables to clarify the pathways by which medication adherence trajectories affect stroke recurrence. This will not only validate our findings but also enhance their relevance for broader clinical applications. Third, while numerous factors influence medication adherence, this study did not account for personality traits or medication-related variables, resulting in a lack of more nuanced insights. Fourth, the incidence of mortality and loss to follow-up during the study contributed to incomplete data points, consequently omitting these cases from trajectory analysis and restricting a comprehensive evaluation of prognosis in CAS patients. Lastly, the single-center design of this study may constrain the generalizability of the findings, highlighting the need for additional research to address these limitations.

## 5 Conclusion

This study is the first to delineate three distinct trajectories of medication adherence among post-CAS patients: “Persistently low level decline” (31.10%), “Medium level decline” (31.58%), and “High level decline” (37.32%). Adherence assessed 3 months post-surgery serves as a predictor for future trajectories, underscoring the critical need for timely interventions within the first 6 months to improve adherence. The high level decline group serves as a crucial protective factor in preventing stroke recurrence. Healthcare providers should adopt early risk prediction and stratification approaches for pharmacological interventions informed by these adherence patterns. This study presents an essential theoretical framework for enhancing post-CAS medication adherence, with significant implications for improving outcomes in this population.

## Data Availability

The original contributions presented in the study are included in the article/Supplementary material, further inquiries can be directed to the corresponding author.
