# Supplementary material for: Medication adherence trajectory and its impact on recurrent stroke after carotid artery stenting
Source: Front Neurol. 2025 Sep 25;16:1637268. doi: 10.3389/fneur.2025.1637268 (PMC12507548; doi:10.3389/fneur.2025.1637268)
Supplement: Supplementary file 1 [file Supplementary_file_1.zip › Supplementary Material/Table_1.DOCX]

**TABLE S1 Evaluation indicators of fitting effect of 1~3 group trajectory models.**

| **_GROUP_** | ***Aveep%*** | **Proportions per class%** | ***π_j_%*** | ***BIC*** | ***△BIC*** | ***OCC*** | ***E_j_*** |
| --- | --- | --- | --- | --- | --- | --- | --- |
| 1Groups(1) | 100.0 | 209(100.0) | 100.0 | -1137 |  |  |  |
| 2Groups(11) | 96.0-95.9 | 82(39.2)-127(60.8) | 40.2-59.8 | -1000 | -137 | 36.2-15.7 | 0.863 |
| 2Groups(22) | 97.1-96.4 | 82(39.2)-127(60.8) | 40.3-59.7 | -950 | -50 | 49.3-18.0 | 0.885 |
| 3Groups(111) | 95.0-87.0-88.5 | 65(31.1)-51(24.4)-93(44.5) | 30.6-27.9-41.4 | -985 | 35 | 43.3-17.3-10.9 | 0.789 |
| **3Groups(222)** | **97.8-87.2-92.3** | **65(31.1)-66(31.6)-78(37.3)** | **31.2-31.1-37.7** | **-917** | **-68** | **98.8-15.2-19.9** | **0.839** |
| 4Groups(1111) | 98.3-97.5-82.5-91.1 | 6(2.9)-61(29.2)-60(28.7)-82(39.2) | 2.8-30.3-27.9-39.0 | -957 | 40 | 2025.2-89.4-12.2-16.0 | 0.849 |
| 4Groups(2222) | 99.8-97.5-88.1-94.9 | 6(2.9)-63(30.1)-69(33.0)-71(34.0) | 2.9-30.3-31.6-35.3 | -873 | -85 | 22393.3-89.5-16.1-34.2 | 0.899 |

**Note:** Good model fit is indicated by the following criteria: (1) Avepp (Average posterior probability) greater than 0.7 for each group; (2) Pj (Posterior probability of group membership) greater than 5%; (3) Close correspondence between Pj and πj (Probability of group membership); (4) BIC (Bayesian Information Criterion) close to 0; (5) A large △BIC (BIC difference between complex and simple models), which supports the acceptance of more complex models; (6) OCC (Odds of Correct Classification) greater than 5; and (7) E (Relative entropy) greater than 0.8.
