# Supplementary material for: Medication adherence trajectory and its impact on recurrent stroke after carotid artery stenting
Source: Front Neurol. 2025 Sep 25;16:1637268. doi: 10.3389/fneur.2025.1637268 (PMC12507548; doi:10.3389/fneur.2025.1637268)
Supplement: Supplementary file 1 [file Supplementary_file_1.zip › Supplementary Material/Table_2.DOCX]

**TABLE S2 Estimation of 3 sets of trajectory model parameters**

| **Group** | **Parmeter** | **Estimate** | **Standard Error** | ***t*** | ***P*** |
| --- | --- | --- | --- | --- | --- |
| Group1 | Intercept | 4.70 | 0.10 | 46.93 | **<0.001** |
|  | Linear | -0.48 | 0.06 | -7.68 | **<0.001** |
|  | Quadratic | 0.033 | 0.01 | 5.14 | **<0.001** |
| Group2 | Intercept | 6.39 | 0.15 | 43.18 | **<0.001** |
|  | Linear | -0.58 | 0.06 | -9.04 | **<0.001** |
|  | Quadratic | 0.05 | 0.01 | 7.09 | **<0.001** |
| Group3 | Intercept | 7.92 | 0.16 | 51.00 | **<0.001** |
|  | Linear | -0.72 | 0.07 | -10.67 | **<0.001** |
|  | Quadratic | 0.058 | 0.01 | 8.645 | **<0.001** |

**Note:** Good model fit is indicated by the following criteria: (1) Avepp (Average posterior probability) greater than 0.7 for each group; (2) Pj (Posterior probability of group membership) greater than 5%; (3) Close correspondence between Pj and πj (Probability of group membership); (4) BIC (Bayesian Information Criterion) close to 0; (5) A large △BIC (BIC difference between complex and simple models), which supports the acceptance of more complex models; (6) OCC (Odds of Correct Classification) greater than 5; and (7) E (Relative entropy) greater than 0.8.
